# Does long COVID in people living with HIV resemble the functional phenotype of non-HIV individuals who had moderate or severe acute COVID-19? A retrospective cross-sectional study

**DOI:** 10.3389/fmed.2025.1533009

**Published:** 2025-04-25

**Authors:** Anna Gabriela Rezende, Jefferson Valente, Cássia da Luz Goulart, Fernanda Facioli, Bernardo Maia, Victor Irungu Mwangi, Hector Bonilla, Frits M. E. Franssen, Camila Miriam Suemi Sato Barros do Amaral, Thaís Jordão Perez Sant'Anna Motta, Marcia Alexandre, Luiz Carlos de Lima Ferreira, Gerson Cipriano Junior, Guilherme Peixoto Tinoco Arêas, Fernando Almeida-Val

**Affiliations:** ^1^Universidade Federal do Amazonas, Manaus, Brazil; ^2^Universidade do Estado do Amazonas, Manaus, Brazil; ^3^Universidade de Brasília, Brasília, Brazil; ^4^Stanford University, Stanford, CA, United States; ^5^Maastricht University Medical Center, Maastricht, Netherlands; ^6^Hospital e Pronto Socorro 28 de Agosto, Manaus, Brazil; ^7^Fundação de Medicina Tropical Dr. Heitor Vieira Dourado, Manaus, Brazil

**Keywords:** HIV, long Covid, functional capacity, pulmonary function, COVID-19

## Abstract

**Introduction:**

It has been postulated that individuals with long COVID have reduced exercise capacity, just as people living with HIV (PLWH), although having even lower exercise capacity. The extent to which long COVID in PLWH resembles long COVID in individuals who presented different COVID-19 phenotypes is unknown, so we aimed to determine if the long COVID profile in PLWH resembled the symptoms experienced by individuals with long COVID following mild/moderate or severe acute COVID-19, 2 years after the initial disease.

**Material and methods:**

A pulmonary function test and a 6-min walk test (6MWT) were performed on adult individuals with PACS (Post-Acute COVID Syndrome) in 3 groups: COVID-19+PLWH (plwHCOV), mild/moderate COVID-19 (mmCOV); severe COVID-19 (seCOV).

**Results:**

Sixty three individuals were included: plwHCOV (*n* = 12), mmCOV (*n* = 33) and seCOV (*n* = 18). Across all groups, males were predominant. BMI was 25 ± 3, 28 ± 4, and 32 ± 7 kg/m^2^ in plwHCOV, mmCOV, and seCOV, respectively (*p* = 0.003). The plwHCOV walked 545 m (±93) at the 6MWT, which was comparable to the mmCOV group (555 m ± 63) but significantly higher than the seCOV group (435 m ± 84) (*p* < 0.0001). The plwHCOV group had worse forced expiratory volume in 1st second (FEV_1_%, 80 ± 12) (*p* < 0.0001), forced vital capacity (FVC%, 83 ± 11) (*p* = 0.002) and FEV_1_/FVC (0.80 ± 0.1, *p* = 0.004) when compared to the seCOV group. Interestingly, PLWH had comparable 6MWT, FEV_1_, FVC, and FEV_1_/FVC results as mmCOV.

**Conclusion:**

Our results indicate that even 2 years post-COVID-19 infection, PLWH exhibits significantly decreased spirometry compared to the seCOV group. Despite this lung function impairment, their functional capacity was similar to individuals with PACS following mild/moderate COVID-19.

## Introduction

COVID-19 and HIV/AIDS are among the most impactful viral diseases of global concern in recent history (1), both of which are known to affect functional and respiratory capacity (2, 3). While most patients recover from acute COVID-19, ~1 in 10 develop long COVID symptoms that can persist for months (4, 5). More than 200 symptoms and conditions, such as postural orthostatic tachycardia syndrome (PoTS) and myalgic encephalomyelitis/chronic fatigue syndrome (ME/CFS), have been associated with long COVID (6). Definitions vary across agencies: the CDC/NIH considers symptoms lasting 4 or more weeks (7), WHO/NICE defines it as symptoms persisting for at least 2 months after 3 months from infection (8), and the RECOVER initiative extends this window to 6 months (6).

Although most long COVID studies focus on prevalence and symptom description in the general population (6, 9–12), fewer have investigated its long-term functional consequences, mainly using objective measures (13, 14). Reduced exercise capacity has been postulated as a hallmark of long COVID (13), yet little is known about how this manifests in specific vulnerable groups.

Globally, nearly 39 million people live with HIV, with rising numbers in Latin America (15, 16). Advances in antiretroviral therapy (ART) have reduced morbidity and mortality (17), but people living with HIV (PLWH) increasingly face challenges such as chronic disease, reduced quality of life, and functional decline linked to viral persistence, frailty, and systemic deterioration (18, 19). Recent evidence suggests that PLWH are at increased risk of developing long COVID, regardless of ART status, CD4 count, or viral load (20, 21). A recent systematic review and meta-analysis further confirmed that HIV infection is associated with a higher likelihood of developing long COVID, with common symptoms including fatigue, cough, and asthenia, and evidence of impaired lung function and immune dysregulation in this population (21).

Despite this elevated risk, the functional profile of PLWH with long COVID remains poorly understood, particularly in comparison to individuals without HIV who experienced different severities of acute COVID-19. Most available studies rely on subjective self-reports, highlighting the need for studies that use objective assessments. Therefore, the present study aims to evaluate the functional profile of PLWH with long COVID in comparison to individuals with long COVID who had mild/moderate or severe acute COVID-19, more than 2 years after initial infection.

## Methods

### Study design and setting

This retrospective cross-sectional study involved 63 individuals of both sexes, over 18 years old, and residents of Manaus. It was conducted at the Federal University of Amazonas (UFAM) in Manaus, Brazil. All individuals had the first confirmed SARS-CoV-2 infection before the vaccine roll-out in Manaus, which started in January 2021.

### Eligibility criteria and study groups

People living with HIV, who had an undetectable viral load (<40 cells/ml), with at least 6 months of adherence to antiretroviral therapy (ART), who had mild/moderate COVID-19 in 2020, and with no history of respiratory or neurological diseases comprised the plwHCOV group, totaling 12 individuals. The comparative groups of people not living with HIV were mmCOV (mild/moderate COVID-19, a total of 33 individuals) and seCOV (severe COVID-19, a total of 18 individuals). The severity of COVID-19 was defined by the provisional clinical guidance of the World Health Organization (WHO) (22). Overall, clinically unstable individuals presenting musculoskeletal, neurological, or respiratory disorders that affected the performance of functional tests were excluded. All subjects had no current or chronic lung disease or health conditions that would interfere with functional and respiratory assessments. The long COVID-19 definition followed the WHO classification (22) and HIV infection was defined as a positive confirmatory molecular test.

### Measurements

Patient clinical data, including age, sex, weight, and height (used to calculate the body mass index, BMI), details on past medical history, comorbidities, persistent symptoms, smoking habits, vital signs, severity of respiratory fatigue by the mMRC (Modified Medical Research Council) dyspnea scale (23) and medication usage were collected to characterize the groups.

#### Pulmonary function and respiratory muscle strength (RMS)

Spirometry (Cosmed^®^, Italy) was performed for lung function and was interpreted according to the *American Thoracic Society (ATS)* and *European Respiratory Society (ERS)* standards. We measured the forced expiratory volume in 1st second (FEV_1_) and the forced vital capacity (FVC). The predicted values were calculated according to the ERS equation (24). The FEV_1_ (L, %), FVC (L, %), and FEV_1_/FVC (%) were recorded (25). Percent-predicted values were determined as per the proposed recommendations by Alberto et al. (26). Maximum inspiratory pressure (MIP) was obtained after the individual expired to residual volume and performed a maximal effort inspiration against a closed valve, during which the pressure was measured. For the maximum expiratory pressure (MEP) assessment, patients underwent an inspiration to total lung capacity, followed by a maximal effort expiration against a closed valve for 2 s, after which the valve was opened (24).

#### Functional capacity

This was determined using the 6-min walk test (6MWT), during which participants received standardized verbal encouragement and instructions to walk the maximum distance possible within 6 min along a flat corridor measuring 30 m in length. At the beginning and end of the tests, vital signs are measured for hemodynamic monitoring: Systolic and diastolic blood pressure (SBP and DBP), heart rate (HR), peripheral oxygen saturation (SpO2), and perception of dyspnea (BORG Scale). The test followed the guidelines set by the *American Thoracic Society* (27). All groups underwent spirometry and 6MWT tests (28).

#### Quality of life - SF-36

The Short Form Health Survey (SF-36) questionnaire was used to assess the Quality of life among individuals living with HIV. This assessment was conducted at an exploratory level, recognizing the challenges related to stigma and social neglect within the PLWH population. The SF-36 evaluates eight quality-of-life domains, which are categorized into physical (functioning, role limitations-physical, pain, general health) and mental health (vitality, social functioning, role limitations-emotional, and emotional/mental health). Scoring was as outlined by Hays et al. (29). Item scores were converted to a scale ranging from 0 to 100 points, where 0 indicated the poorest perception of health, and 100 reflects the best perception of health. Averaging individual items within the subscale and physical composite and mental health determined the domain scores.

### Ethical aspects

The Federal University of Amazonas Institutional Review Board approved this study (CAAE 44971221.7.0000.5020). This adhered to the principles outlined in the Declaration of Helsinki and the Good Clinical Practice guidelines of the International Conference on Harmonization. Eligible before enrollment. They were allowed sufficient time to thoroughly review and sign an informed consent form (ICF).

### Statistical analysis

The Shapiro-Wilk and Levene's tests assessed data normality and homogeneity, respectively. The results were presented as mean ± standard deviation, median, and interquartile range, or percentage values. The Chi-square test and ANOVA one-way test *post hoc* Tukey test were used for group characteristic analyses. Pearson's correlation determined the correlation coefficient between variables, considering indices from 0.1–0.3, 0.4–0.6, and 0.7–0.9 with weak, moderate, and strong associations, respectively (30). A *p*-value < 0.05 was considered statistically significant. The SPSS software version 23.0 (IBM, Chicago, USA) was used for calculations, and GraphPad Prism software version 8.0 (GraphPad, California, USA) was used for creating the images. The sample size was calculated using G^*^Power 3.1 software for a Pearson correlation analysis, assuming a moderate effect size (*r* = 0.5), a significance level of α = 0.05, and a statistical power (1–β) of 0.80. Based on these parameters, the minimum required sample size was estimated at 29 participants. The actual number of participants included in each subgroup provided slightly higher statistical power (~86%), reducing the likelihood of a type II error.

## Results

### Population characteristics for the plwHCOV group

Participants from the plwHCOV group were predominantly male, above 40 years of age, living with HIV for at least 12 months, and adherent to ART. For a detailed individual description of this group, please see Table 1. Clinical, functional, and lung function characteristics of individuals are detailed in Table 2. The most frequent comorbidity in the plwHCOV group was hypertension, while the most reported medication used daily was Losartan. All participants denied engaging in harmful habits and addictions, as described in Table 1. The FEV_1_ was 2.6 ± 0.7 L, FEV_1_ predicted 80 ± 12%, FVC 3.3 ± 0.9 L, FVC predicted 83 ± 11%, FEV1/FVC 0.80 ± 0.1 L/s, MIP 92 ± 39 cmH2O, MEP 105 ± 33 cmH_2_O. The SF-36 mean total score was 51.9 ± 29.4. Details of the quality-of-life assessment are also available in Table 1.

**Table 1:** Sociodemographic, HIV status and quality of life characteristics of the PLWHCOV group.

**ID**	**Sex/Age**	**BMI**	**Years with HIV**	**CD4 levels**	**Comorbidities**	**Quality of life (SF-36 questionnaire)**								
						**Physical function**	**Role physical**	**Role emotional**	**Vitality**	**Mental health**	**Social function**	**Bodily pain**	**General health**	**Total score**
1	M/48	26.8	9y	603	Hypertension	100	100	0	60	68	87.5	57.5	65	62.7
2	F/51	24.6	12y	678	Hypertension	35	25	100	0	76	0	45	30	38.8
3	F/46	24.2	23y	772	No	35	0	0	15	20	25	35	35	20.6
4	F/53	18.9	12y	602	No	55	0	0	5	4	37.5	22.5	40	20.5
5	F/51	28.7	8y	1,012	Depression	15	0	0	55	48	25	10	10	20.3
6	M/35	25.9	1y	771	Rheumatoid arthritis	40	0	0	25	40	12.5	22.5	30	21.2
7	M/26	24.2	2y	542	No	100	100	100	75	84	87.5	100	60	88.3
8	M/44	28.4	7y	481	No	100	100	100	90	76	25	57.5	75	77.9
9	M/31	24.1	7y	1,107	Anxiety	100	100	0	50	52	75	90	75	67.7
10	M/40	23.3	8y	676	No	85	0	0	15	40	0	45	55	30
11	M/36	28.3	7y	637	No	100	100	100	55	72	75	100	55	82.1
12	M/42	23.8	7y	1,090	No	100	100	66.6	100	84	100	100	95	93.2
						72 ± 33^*^	52 ± 50^*^	38 ± 48^*^	45 ± 33^*^	55 ± 25^*^	45 ± 36^*^	57 ± 33^*^	52 ± 23^*^	51.9 ± 29.4^*^

M, Male; F, Female; BMI, Body Mass Index in kg/m^2^; Y, years; CD4 in cells/mm3; ART, Antiretroviral Therapy; DTG, Dolutegravir; 3TC, Lamivudine; TDF, Tenofovir; EFZ, Efavirenz; DRV, Darunavir; RTV, Ritonavir; ^*^Mean score of each domain, Mean ± SD.

**Table 2:** Clinical, functional, and lung function characteristics of individuals with COVID.

**Variables**	**plwHCOV (*n =* 12)**	**mmCOV (*n =* 33)**	**seCOV (*n =* 18)**	***P* value**
**Age (years)**	42 ± 8	42 ± 13	46 ± 7	0.437
Sex, *n* (%)				
Male	8 (67)	19 (58)	14 (78)	**0.028**
Female	4 (33)	14 (42)	4 (22)	
**BMI, kg/m** ^**2**^	25 ± 3^*^	28 ± 4^#^	32 ± 7	**0.003**
mMRC score				
0	2 (17)	18 (55)	4 (22)	**<0.0001**
I	4 (33)	14 (42)	8 (44)	
II	6 (50)	1 (3)	6 (33)	
6MWT				
Walking distance (m)	545 ± 93^*^	555 ± 63^#^	453 ± 84	**<0.0001**
% predicted of 6MWT	81 ± 28^*^	100 ± 14^#^	79 ± 18	**<0.0001**
Hemodynamic variables of the 6MWT				
Initial SBP (mmHg)	118 ± 9	111 ± 15^#^	106 ± 45	0.459
Initial DBP (mmHg)	79 ± 7	78 ± 10	76 ± 34	0.938
Final SBP (mmHg)	129 ± 17^*^	123 ± 18^#^	100 ± 50	**0.022**
Final DBP (mmHg)	85 ± 10	78 ± 9	68 ± 32	0.058
Initial HR (bpm)	74 ± 8	81 ± 12	73 ± 19	0.101
Final HR (bpm)	83 ± 7 ^a*^	127 ± 22^#^	84 ± 34	**<0.0001**
Initial SpO_2_ (%)	96 ± 1	96 ± 4^#^	92 ± 23	0.560
Final SpO_2_ (%)	97 ± 1	97 ± 1	81 ± 37	**0.026**
Initial BORG dyspnea	1.5 ± 1	0.9 ± 0.9^#^	1.9 ± 1.8	**0.049**
Final BORG dyspnea	3 ± 1	2.5 ± 1.4	3.0 ± 1.9	0.616
Spirometry				
FEV_1_, L/s	2.6 ± 0.7^a^	3.1 ± 0.7^#^	2.6 ± 0.6	**0.038**
FEV_1_, %	80 ± 12^a*^	98 ± 10^#^	94 ± 14	<0.0001
FVC, L/s	3.3 ± 0.9	3.8 ± 0.9	3.2 ± 0.6	0.076
FVC, %	83 ± 11^a*^	98 ± 10	94 ± 14	**0.002**
FEV_1_/FVC, L/s	0.80 ± 0.1^*^	0.87 ± 0.1	0.94 ± 0.1	**0.004**
Respiratory muscle strength				
MIP (cmH_2_O)	92 ± 39	100 ± 36	88 ± 30	0.509
Inspiratory muscle weakness	**2 (16)**	6 (18)	2 (11)	0.800
MEP (cmH_2_O)	105 ± 33	122 ± 47	95 ± 40	0.095
**HIV time (years)**	8 ± 5	–	–	–

plwHCOV, HIV+COVID-19; mmCOV, mild/moderate COVID-19; seCOV, severe COVID-19.

^*^p < 0.05 plwHCOV vs. seCOV; ^#^p < 0.05 seCOVvs mmCOV; ^a^p < 0.05 plwHCOV vs mmCOV. BMI, Body mass index; mMRC, modified-Medical research council;

6MWT, 6-min walk test; SBP, Systolic blood pressure; DBP, Diastolic blood pressure; SpO2, O2 saturation; BORG, Scale for dyspnea/fatigue;

FEV, Forced expiratory volume; FVC, Forced vital capacity; MIP, Maximum inspiratory pressure; MEP, Maximum expiratory pressure.

Bold values indicate statistical significance.

**Figure 1:**
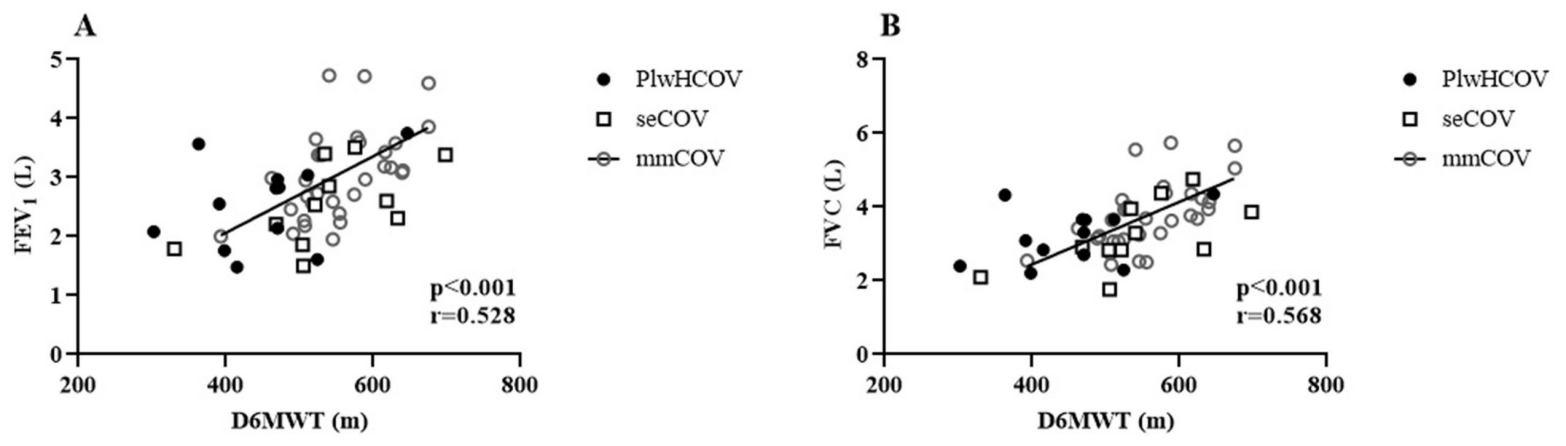
**(A)** Distance Walked in the 6-Minute Walk Test (D6MWT). **(B)** Maximum Expiratory Pressure (MEP).

In the plwHCOV group, we found strong correlations (Figure 1) between D6MWT and MEP (*r* = 0.700, *p* = 0.016) and a moderate correlation between SF-36 total score and MEP (cmH_2_O) (*r* = 0.633, *p* = 0.027), demonstrating that respiratory muscle strength directly affects the functional capacity and quality of life.

### Population characteristics for the mmCOV and seCOV groups

The mmCOV group had an average age of 42 years and a more balanced gender distribution (58% were male). The mean BMI was 28 kg/m^2^. This group had the best functional performance, covering the longest distance in the 6MWT (555 ± 63 m), reaching 100% of the predicted value. FEV1 (98%) and FVC (98%) were the highest among the groups, indicating better lung function. In the seCOV group, the average age was 46 years, with a male predominance (78%) and the highest BMI (32 kg/m^2^). Functional capacity was the most impaired, with the shortest 6MWT distance (453 ± 84 m, 79% predicted). Lung function was observed with an FEV1 of 94% and FVC of 94%, along with the highest FEV1/FVC ratio (0.94).

### Functional assessment outcomes

The functional assessments in this study were conducted on average 24 ± 5 months after the initial acute COVID-19 in all groups, and all were done before the COVID-19 vaccination roll-out. The proportion of mMRC II scores across groups was 6 (50%), 1 (3%), and 6 (33%) in plwHCOV, mmCOV, and seCOV, respectively. The male sex was predominant across all groups. The plwHCOV group had worse forced expiratory volume in 1st second (FEV_1_%, 80 ± 12) (*p* < 0.0001), forced vital capacity (FVC%, 83 ± 11) (*p* = 0.002) and FEV_1_/FVC (0.80 ± 0.1, *p* = 0.004) when compared to the seCOV group. Interestingly, the plwHCOV group responded similarly to the mmCOV group for the 6MWT, FEV_1_, FVC, and FEV1/FVC (Table 2).

### Correlation analysis

**Figure 2:**
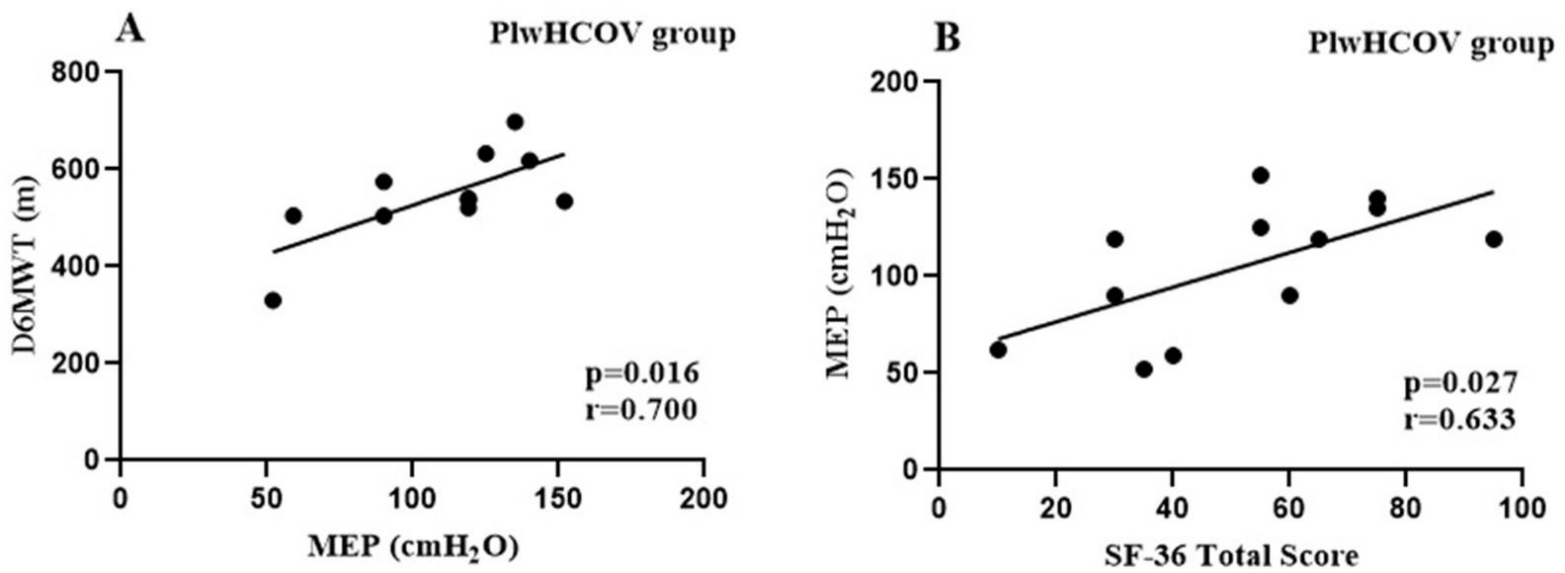
**(A)** Forced Expiratory Volume in One Second (FEV1). **(B)** Forced Vital Capacity (FVC).

We found a moderate correlation association between the walked distance in the 6MWT (6MWD) and FEV_1_ (L) (*r* = 0.528, *p* < 0.001) and D6MWT and FVC (L) (*r* = 0.568, *p* < 0.001) (Figure 2) in all three groups. All correlations were tested within the individual populations. For the groups without HIV, the correlation results were: seCOV (*p* = 0.001, *r* = 0.60) and mmCOV (*p* = 0.041, *r* = 0.61).

## Discussion

This study showed a significant proportion of individuals living with HIV presenting significant functional and Quality of life deficits long after COVID-19 infection. Comparing the clinical profiles of long COVID between PLWH and individuals who did not live with HIV and had moderate and severe COVID-19, we observed that PLWH presented functional capacity parameters like moderate cases. At the same time, their lung function was worse than individuals who had had severe COVID-19. Furthermore, we observed correlations between respiratory parameters with D6MWT and Quality of life in the group of individuals living with HIV.

The majority of PLH individuals in this study were male and aged above 40 years, similar to other studies (31–36). Here, individuals living with HIV demonstrated a 6MWT walked distance above the average of 400 m previously reported for this population (34). Studies assessing long COVID using the 6MWD outcome report values ranging from 400 to 500 m (13, 37–40) The progression of long COVID-19 is associated with reduced exercise capacity and physical activity levels, which may exacerbate exercise intolerance and ultimately lead to a decline in functional capacity and the ability to perform activities of daily living (41). In this study, the plwHCOV and mmCOV groups showed greater 6MWT distances compared to the seCOV group. Previous studies have shown that severe acute COVID-19 is characterized by more intense symptoms, greater systemic involvement, and longer hospitalization and recovery periods; thus, worse post-illness conditions were expected (11, 37, 42–45). For instance, researchers assessing functional deficiencies post-COVID-19 using the 6MWT in a Mexican population aged above 40 years found that individuals with moderate to severe disease walked shorter distances than those with mild cases, with this decline independently associated with breathing difficulties and respiratory function changes (46). The extent to which the overlap between long COVID and HIV, or the impact of hospitalization and invasive support during severe and critical acute COVID-19, contributes to functional capacity decline in these populations remains largely unknown and warrants further research.

The plwHCOV group also showed reduced lung function compared to the mmCOV group. Previous studies have demonstrated that PLWH may experience pulmonary deficits associated with both HIV itself and opportunistic respiratory conditions (47). In our sample, hypertension was the most frequent comorbidity among PLWH, and losartan was the most commonly used antihypertensive. Although some experimental and preclinical studies have suggested a potential protective effect of losartan against lung fibrosis and inflammation through TGF-β modulation and attenuation of AngII activity (48) these findings have not been supported by robust clinical evidence. A multicenter randomized controlled trial in hospitalized patients with COVID-19 showed that losartan did not improve oxygenation, severity of illness, or other clinical outcomes and even raised concerns about potential adverse effects on hemodynamics and renal function (49). Similarly, a large placebo-controlled trial evaluating losartan in patients with emphysema found no benefit in preventing disease progression or improving pulmonary function (50). Based on this high-quality evidence, it is unlikely that losartan biased pulmonary outcomes in our PLWH group; if anything, its use may have attenuated even poorer results. Importantly, participants in the mmCOV and seCOV groups did not report hypertension or use of losartan, which is shown in the results section to address potential treatment-related confounding.

We also observed a correlation between respiratory muscle strength and the distance walked among PLWH participants, suggesting that respiratory performance directly influences functional capacity, as previously described in the literature (2, 51–53). Collini (54) showed that PLWH on antiretroviral therapy experience faster pulmonary decline than HIV-negative individuals, driven largely by systemic inflammation (55–58). This chronic inflammation, combined with the known respiratory consequences of COVID-19 (22, 59–61), may help explain the reduced lung function observed in the plwHCOV group. The relationship between pulmonary function and functional capacity is well established (38), and our findings reinforce this association, with significant correlations observed between spirometric values and 6MWD across all groups.

Studies have reported that PLWH has lower-than-expected perceptions of Quality of life due to multifactorial factors (62–65); among them, non-use or irregular use of ART was indicative of worse perceptions of Quality of life (66–68). In this study, although the individuals were all on regular ART, their perceptions of Quality of life in the SF-36 questionnaire were below that established in the literature (69). They significantly correlated with the individuals' expiratory muscle strength. Previous studies evaluating respiratory muscle strength in PLWH indicated the influence of respiratory parameters on the general condition of individuals, in addition to the relationship between adequate respiratory muscle strength and Quality of life in other health contexts already established in the literature (19, 70, 71).

Finally, although BMI differed significantly across groups—with higher values in the seCOV group—the spirometry outcomes were expressed as predicted percentages adjusted for age, sex, and height, which minimizes the influence of body composition on interpretation. Moreover, the group with the lowest BMI (plwHCOV) exhibited the most reduced spirometric parameters, suggesting that BMI alone was not the primary factor influencing pulmonary function in this cohort. Nonetheless, we acknowledge that excess body weight may contribute to a restrictive ventilatory pattern in some individuals, particularly within the seCOV group, and this potential influence cannot be entirely excluded.

Our study has some limitations. Due to its cross-sectional design, the study cannot establish causal relationships between the observed outcomes and prior COVID-19 or long COVID. It is therefore unclear whether the reduced functional or respiratory measures observed, particularly in individuals with long COVID, were pre-existing or developed as a consequence of the infection. Additionally, no pre-COVID-19 data on lung function or functional capacity were available for comparison. The relatively small sample size and the fact that this was a single-center study may also limit the generalizability of the findings. Participants were heterogeneous in terms of BMI, which may have influenced the respiratory outcomes. The plwHCOV group was particularly limited in size due to challenges in recruiting individuals living with HIV, many of whom expressed concerns about confidentiality—an issue often driven by persistent social stigma. These concerns also motivated the inclusion of quality of life assessments in this group.

## Conclusion

People living with HIV/AIDS showed worse lung function compared to individuals without HIV but had severe COVID-19, yet showed similar results in 6MWT, FEV_1_, FVC, and FEV_1_/FVC as those with moderate/mild COVID-19. Our findings highlight persistent deficits in lung function among PLWH even after more than 2 years post-COVID-19 infection, significantly impacting their functional capacity and Quality of life. Targeted interventions involving cardiorespiratory and motor rehabilitation are essential for PLWH experiencing long COVID. Further comprehensive studies are needed to comprehend the contribution of HIV to functional deterioration in long COVID cases.

## Data Availability

The raw data supporting the conclusions of this article will be made available by the authors, without undue reservation.
